# Immediate Postoperative Portable Radiograph After Total Knee Replacements: A Necessity or a Burden?

**DOI:** 10.2174/1874325001812010173

**Published:** 2018-05-31

**Authors:** Abdulla Aljawder, Dana Alomran, Mohammed Alayyoub, Fahad Alkhalifa

**Affiliations:** 1 Bahrain Defense Force Royal Medical Services, Orthopedic Specialist, Orthopedic Department, Alriffa, Bahrain; 2 Bahrain Defense Force Royal Medical Services, Orthopedic Resident, Orthopedic Department, Alriffa, Bahrain; 3 RCSI-MUB, Collage of medicine, Al Muharraq, Medical student, Bahrain; 4 Bahrain Defense Force Royal Medical Services, Senior Orthopedic Consultant, Orthopedic Department, Alriffa, Bahrain

**Keywords:** Total knee replacement, Radiograph, Arthroplasty, Postoperative, Recovery room, Portable

## Abstract

**Background:**

Total Knee Replacement (TKR) is one of the most commonly performed orthopaedic surgery(1). Immediate postoperative portable radiographs are performed after primary TKR in order to identify any potential complications and technical flaws. It also serves as a reference for comparison with subsequent radiographs. The aim of this study was to evaluate the clinical and economical value of these radiographs in TKR. It compares the quality of the portable radiograph, taken immediately post-operation, with in-suite radiographs taken 5-7 weeks post-operation

**Methods:**

In this retrospective study, a consecutive series of 389 TKR patients from January-2011 to March-2015 were reviewed. Radiological evaluation consisted of assessing the beam angle and the exposure on the images. Implant positioning was also compared by measuring the anatomical axis to look for component alignment discrepancies.

**Results:**

The quality of the portable recovery room radiograph was overall inferior to the radiology suite radiograph regarding both beam angle and exposure. Component alignment discrepancies were also identified in the angle measurements between both types of radiographs.

**Conclusion:**

Therefore, our study demonstrated that there is no clinical or financial value obtained from postoperative portable radiograph. Furthermore, Immediate recovery room radiographs should be avoided from being performed routinely and may only be used in cases where the surgeon is utilizing a new implant or technique. No external funding was provided for this study from any source.

## 1 INTRODUCTION

Total knee replacement is one of the most commonly performed orthopaedic surgeries [1, 2]. The purpose of the procedure is to improve the biomechanics of the knee joint by replacing the damaged joint with a prosthetic implant, realigning the soft tissues, and eliminating structural as well as functional deficits [3]. Due to its high success rates and the aging population, the number of total knee replacements annually is expected to rise in the upcoming years [2]. This entails an expected increase in healthcare expenditure. The practice of cost effective care has become a priority in virtually all healthcare institutions. In order to do so, a cost containment strategy must to be implemented in order to ensure that the increase in the number of total knee replacements will not burden the healthcare budget while maintaining the quality of care.

Immediate postoperative portable radiographs are commonly performed after primary total knee replacements in several healthcare institutions [4]. This is a routine practice in our center as well. The purpose of these radiographs is to identify any potential complications or technical flaws that may alter the immediate management of the patient. The surgeon uses these images to inspect for fractures, dislocation, implant orientation, and retained drains. They are also used as a reference for comparison with subsequent radiographs and to recommend further diagnostic investigations when needed. Postoperative portable radiographs are often taken in the recovery room where conditions are usually not ideal for proper positioning [5].The necessity of these portable radiographs has been under scrutiny in several studies [5-7]. They may lead unnecessary radiation exposure to the patient and healthcare workers. The aim of this study was to evaluate the clinical and economic value of immediate postoperative portable radiographs in total knee replacement by comparing the quality of the portable radiographs with the radiographs taken in the radiology suite. Furthermore, we determined if any changes to the management of the patient were made in consequence of the portable radiographs as well as the associated healthcare costs of these investigations.

## 2 MATERIALS AND METHODS

In this retrospective study, a consecutive series of 389 patients who underwent total knee replacement from January 2011 to March 2015 were reviewed. All patients who have performed the surgery were included in this study regardless of the surgical indication. All of these patients had an immediate post-operative portable radiographs in the recovery room taken by radiology technicians. At follow-up, 5 to 7 weeks postoperatively, another radiograph was taken in the radiology suite. We compared the images taken by the portable radiographs and the radiology suite in terms of quality. Radiological assessment was accomplished by assessing the beam angle and the exposure of both Anteroposterior (AP) and lateral projections [8]. Measuring the clear space between the tibial tray and the femoral component was used to assess the beam angle accuracy. The minimum thickness of polyethylene inserts manufactured are 6 mm thick under the femoral condyle in order to resist wear [4, 18]. The beam angle was considered excellent if more than 6 mm, good if between 3 to 6 mm, fair if measuring between 1 and 3 mm, and poor if the image showed overlapping components or if a component was excluded from the film. Exposure was rated as being adequate if the observer can easily distinguish the medullary bone from the cortical bone and when there is sufficient contrast between the bone-cement interfaces. The image exposure was considered underpenetrated if the image was not adequate and over penetrated if diffusely dark.

The images were also assessed for their capability of evaluating implant positioning [8-10]. The component alignment angle of the knee implant was measured for both the femoral component and the tibial component in both the AP and the lateral views. The femoral component and the tibial component coronal alignment was measured on the portable and radiology suite radiographs in the AP views. Femoral alignment was calculated by drawing a tangent to the distal femoral condyles and a line along the femoral anatomical axis and then measuring the angle subtended. Tibial alignment angle is calculated by drawing a tangent across the tibial base plate and drawing a line along the anatomical axis of the tibia [11-14]. The purpose of measuring the anatomical axis was to determine if the images of the portable radiographs could provide measurements as accurate as those provided by the images taken in the radiological suite. The anatomical axes of the tibia and femur were drawn as a line best bisecting the respective medullary cavities. Next we reviewed all the patients’ records to identify if any changes were made in their management plan based on findings discovered in the postoperative portable radiographs. This was completed to evaluate the necessity of the immediate postoperative portable radiographs. The same surgeon in our center performed all total knee replacements. The prostheses used in our total knee replacements were the Genesis and Genesis 2 (Smith & Nephew, London, United Kingdom). The brands of the portable radiography machines were Siemens mobile tt XP digital (Digital film, Settings: 56 kVp, 5 mAs) and GE optima Hualun Medical Systems (Digital Film, Settings: 70 kVp, 4 mAs). Both these devices required the operator to adjust the distance from the patient’s knee. In the radiology suite the machines were General Electric (Digital Film, Settings: 70 KVp, 5.0 mAs, 250 mA, 20 msec) and Siemens axiom Aristos FX plus (Digital Film, 63 KVp, 5mAs, 500 mA). Here the radiographs were performed in a supine position with a fixed distance of 100 cm from the patient’s knee. All images had 15% magnification. The cost of the post-operative radiographs was obtained from our hospital’s cost-billing department. Transport services, x-ray technician time, repeated radiographs, and maintenance of radiographic equipment were not included in the cost. Ethical approval was obtained from the Bahrain Defense Hospital Ethics committee. No external funding was provided for this study from any source. The computer program IBM Statistical Package for Social Sciences (SPSS, Version 20) was used for statistical analysis and box plots. All the radiographs reviewed in this study were analysed using the digital version of the radiographs *via* the software JiveX [rv] Review client 4.4.3.The analysis was performed by two orthopaedic surgeons in our hospital thereby attempting to negate possible inter-observer variability.

## 3 RESULTS

**Fig. (1):**
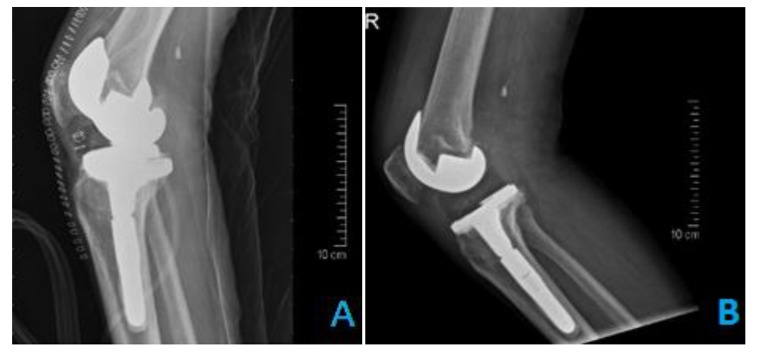
A comparison of the recovery room radiographs (A) *vs.* radiology suit radiograph (B) in the same knee demonstrates an inferior quality of the recovery room radiograph (A).

**Fig. (2):**
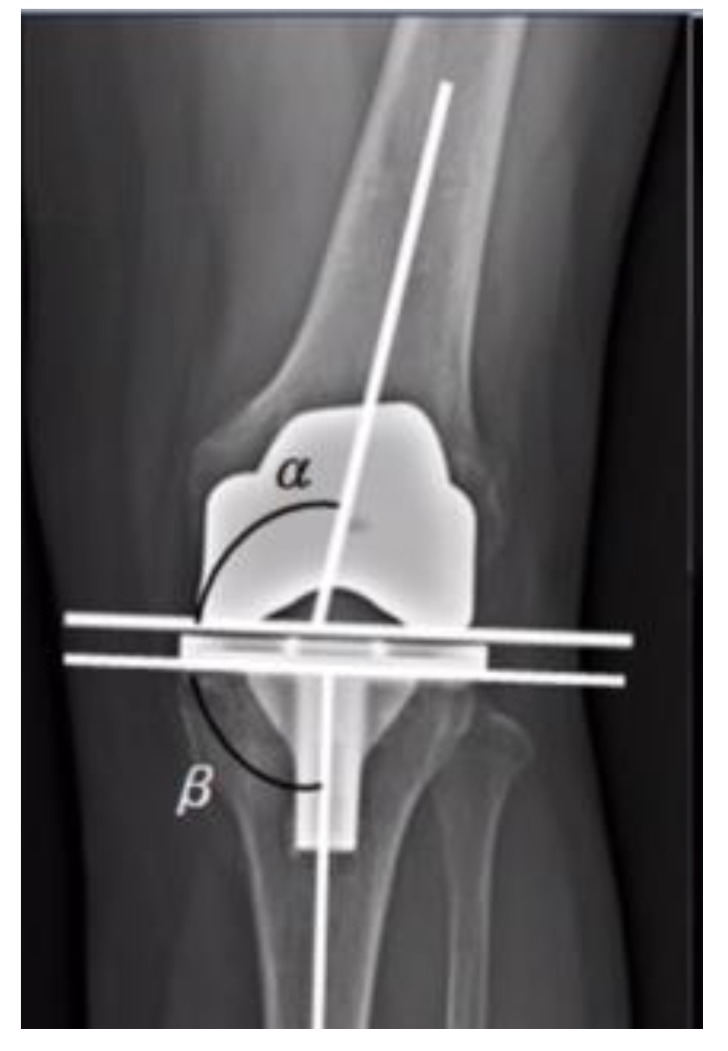
Methods of coronal angle measurements on AP views. Femoral component coronal alignment = 90 - alpha Tibial component coronal alignment = 90 - beta

Radiographic analysis was completed for 389 patients. The mean age was 64 years (range 35-89). Of these 382 patients, 99 (25.7%) were male and 283(73.5%) were female. 7 patients were excluded from the study because they had no follow up radiographs .The quality of the portable recovery room radiographs were overall inferior to the radiology suite radiographs regarding both beam angle and exposure (Tables **1** and **3** / Figs. **1** and **2**). In terms of exposure, all (100%) the radiographs of the radiology suite were adequate while more than (40%) of the portable recovery room radiographs were considered inadequate (Table **3**). Concerning the beam angle accuracy in the AP view, 91% of the radiology suite radiographs were classified as excellent while only 54% of the portable recovery room radiographs were classified as excellent (Table **1**). And with regards to the beam angle accuracy in the lateral view, 84% of the radiology suite radiographs were classified as excellent while only 30% of the portable recovery room radiographs were classified as excellent (Table **1**). More than 30% of the portable recovery room radiographs were classified as poor in terms of beam angle accuracy in the lateral view (Table **1**). Component alignment discrepancies were identified in the angle measurements between the radiology suite radiographs and the portable recovery room AP radiographs (Table **2**). The mean difference was 1.83508 degrees of a more valgus alignment of the femoral component (p <0 .001) in the recovery room radiographs and 2.48429 degrees of a more varus alignment of the tibial component alignment (p <0 .001) in the recovery room radiographs (Table **2**). Out of the 389 patients reviewed, none had their management altered due to findings on the recovery room radiographs. There were no gross complications requiring immediate intervention identified in the postoperative recovery room radiographs. The cost of the post-operative radiographs was approximately 59.95 USD per patient totaling to 23,321 USD over the study period. This figure represents the cost of the film only. It does not include transport services, x-ray technician time, repeated radiographs, and maintenance of radiographic equipment.

## 4 DISCUSSION

Obtaining postoperative portable radiographs after total knee replacement is the standard of care in many healthcare institutions – often completed in the recovery room [5]. The purpose of these radiographs is to provide instant feedback regarding fractures, implant orientation, dislocation, and retained drains of the knee. In theory, the use of these portable radiographs would seem a valuable asset in determining any potential error that maybe missed during the procedure. However, in practice many studies have demonstrated the use of these radiographs after total knee replacement provides little in the management of the patient. Major operative errors that are not identified intraoperatively seldom occur; particularly if the surgery was performed by a trained arthroplasty surgeon. Mechanical failures occurring immediately following primary total knee replacements are a rare occurrences [5-8]. In our institution postoperative radiographs are taken in the recovery room as opposed to in the operating room to avoid disrupting theatre traffic and minimize operative time. Lead aprons are routinely not worn during this procedure and this may lead inadvertent contamination as theatre staff move about to avoid radiation exposure. The involved surgeon does not prefer radiology suite radiographs due to the long wait until the patient fully recovers and can be safely transferred. The radiographs are taken on the recovery trolleys, with the leg in full extension and neutral rotation for the AP view. For lateral radiographs the hip is externally rotated and the knee flexed to approximately 45 degrees of flexion.

Here we compared the quality of the postoperative portable recovery room radiographs with the radiology suite radiographs. The results demonstrated that the quality of postoperative portable radiographs were far inferior and unreliable. After reviewing the images of 382 patients, exposure in 40% of the recovery room radiographs were largely inadequate for analysis. On the other hand, all radiographs of the radiology suite were deemed to be adequate. The beam angle accuracy in the AP view of the portable radiographs were satisfactory as most of the images were in the “excellent” or “good” category and only 2% of the images were categorised as being poor. However, the beam angle accuracy in the lateral view of the portable recovery room radiographs was poor in 30% rendering them unreliable for assessment. The component alignment was our main tool to evaluate the informative value obtained from both radiographs. To note the normal values of the femoral component was taken to be 5 to 9 degrees of valgus alignment relative to the long axis of femur [15]. Also normal reference values of the tibial component alignment was taken to be 90±3 degrees to the long axis of tibia [16]. Our analysis revealed that the angles measured in portable recovery room radiographs were different from those measured in the radiology suite. The mean difference between the two radiographs was 1.83508˚ valgus (CI 1.52255, 2.14671) for the femoral component and 2.48429˚ varus (CI 2.22942, 2.73917) for the tibial component. Furthermore, 107 out of 382 patients had femoral component alignment angles above the acceptable range (5-9 degrees) in the recovery room radiographs but were found to be in the normal range when measured on the radiology suite radiographs. Similarly, 364 out of 382 patients had tibial component alignment angles outside the acceptable range (90±3 degrees) in the portable radiographs but were normal when measured on the radiology suite radiographs. These results show that immediate postoperative portable radiographs are inaccurate in assessing the implant alignment and may raise a false alarm. Next we determined whether any changes in the immediate management of the patients were made as a consequence of the postoperative portable radiographs. None of our patients suffered from complications or errors requiring immediate attention, alteration in treatment plan, or return to the operating room. Our findings demonstrate that there are no added benefits in using the portable radiographs as they do not add value to the management of the patient. The conditions in the recovery room are often suboptimal. This plays a major role in the quality of the portable radiograph obtained. The knee of the patient is usually not fully extended due to postoperative pain and bulky dressing and this has been demonstrated to increase apparent valgus angulation drastically [17]. Also the distance of the x-ray source from the knee when the images are taken is operator dependent with portable radiographs. Varying distances make portable radiographs unreliable to be used as a reference for subsequent radiographs. This correlates with a number of reports that proved that the quality of these portable radiographs is questionable.5,6,8 Glaser and Lotke demonstrated in their study that just 36% of the portable radiographs were adequate.5 Complete Visualization of the knee during TKA negates the need to assess the knee again immediately using the portable radiograph . This raises the question whether the radiation exposure to healthcare professionals and patients as well as the cost of these radiographs is justified. This study was the first objective evidence of the poor quality of recovery room radiographs being performed following this procedure. It has subsequently altered our clinical practice. Our study demonstrates that there is no clinical or financial value obtained from postoperative portable radiograph. The cost of the postoperative portable radiographs at our institution is 59.9775 USD and approximately 100 TKAs are performed annually. Eliminating the use of routine portable recovery room radiographs could save an estimated 6000 USD annually in hospital expenditures. We propose the use of radiology suite postoperatively when the patient is discharged from the hospital and following removal of the dressing. Immediate recovery room radiographs should be avoided from being performed routinely. However, portable radiographs may be used in cases where a surgeon wants to rule out iatrogenic fractures or major failures if not directly visualized intraoperative. The use of radiographs should also be delayed until the first office visit; this will not compromise the quality of care as proven by Moskal and Diduch [14]. Our study had several limitations. A digital PACS system (JiveX [rv] Review client 4.4.3) was used to execute the measurements, but this large involved user judgement and may predispose to human error. The possibility that our results may only apply to the prosthesis used in our study may also be a potential limitation. Also the experience, education, and proficiency of the radiographers were not assessed for both the recovery room and the radiology suite radiographs. This may have implications to interpreting our results; though in our center the same group of radiographers perform both of these investigations. The orthopaedic surgeons were not blinded to the study hypothesis while reviewing and analysing the subjects, which may introduce bias to the study and underestimate the importance of obtaining postoperative portable radiographs. Furthermore this data cannot be extrapolated to all types of joint replacements as only total knee replacements were evaluated. We advise further studies evaluating the usage of immediate postoperative portable radiographs in other total joint replacements surgeries.

## CONCLUSION

Postoperative portable radiographs are commonly performed in several health institutions immediately after knee arthroplasty surgery. The aim of this is to identify complications and technical flaws that would require immediate intervention. This is an extremely rare occurrence in the hands of a well-trained arthroplasty surgeon. Furthermore our study revealed that a large proportion of the portable radiographs taken were deemed inadequate for proper assessment, adding even less to the management of these patients. Whilst, those taken in the radiograph suite prior to the first outpatient visit provide sufficient information for technical analysis. In hand with the current era of evidence driven medical practice and economical health care solutions we advocate that these radiographs be omitted from being routinely performed following total knee replacement surgeries. However, intraoperative portable radiographs can also be obtained electively in order to exclude intraoperative fractures or major operative failures once suspected.

## Figures and Tables

**Table 1:** Beam angle accuracy.

**Scoring System **	**Ap View**		**Lateral View**	
–	**RRR**	**RS**	**RRR**	**RS**
Excellent: > 6 mm	206 (54%)	349 (91%)	115 (30%)	321 (84%)
Good: 3-6mm	154 (40%)	30(8%)	94 (25%)	39 (10%)
Fair: 1-3 mm	15 (4%)	1 (0.5%)	52 (13%)	3 (1%)
Poor : overlapping or if a component was excluded from the film	7 (2%)	2 (0.5%)	121 (32%)	19 (5%)
**P Value**	( p <0 .001 )*		( p <0 .001 )*	
**Correlation coefficient**	(0.295)		(0.521)	

mm: millimetres *A p value of 0.01 or less is significant

**Table 2:** AP radiograph alignment.

**Type**	**Mean**		**Mean** **Difference**	**Confidence** **Interval**
–	**RRR**	**RS**	–	–
FEMORAL COMPONENT ALIGNMENT	7.9974˚ Valgus	6.1623˚ Valgus	1.83508˚ Valgus	(1.52255, 2.14671)
**P Value**	( p <0 .001)*	–	–	–
**Correlation coefficient**	(-0.385)	–	–	–
TIBIAL COMPONENT ALIGNMENT	3.9843˚ Varus	1.5˚ Varus	2.48429˚ Varus	(2.22942, 2.73917)
**P Value**	( p <0 .001)*	–	–	–
**Correlation coefficient**	(-0.570)	–	–	–

*A p value of 0.01 or less is significant

**Table 3:** Exposure.

–	**RRR**	**RS**
Adequate	234 (61%)	382 (100%)
Underpenetrated	126 (33%)	0
Overpenetrated	22 (6%)	0
**P value**	( p < 0.001 )*	–
**Correlation coefficient**	(0.463)	–

*A p value of 0.01 or less is significant

## LIST OF ABBREVIATIONS

AP: Anteroposterior
TKR: Total Knee Replacement
